# The interaction effects between TLR4 and MMP9 gene polymorphisms contribute to aortic aneurysm risk in a Chinese Han population

**DOI:** 10.1186/s12872-019-1049-8

**Published:** 2019-03-29

**Authors:** Tan Li, Xu Zhang, Liang Sang, Xin-tong Li, Hai-yang Sun, Jun Yang, Yuan Yuan

**Affiliations:** 1 Tumor Etiology and Screening Department of Cancer Institute and General Surgery, The First Hospital of China Medical University, No.155 Nanjing Bei Street, Heping District, Shenyang, Liaoning Province People’s Republic of China 110001; 2 Department of Cardiovascular Ultrasound, the First Hospital of China Medical University, Shenyang, 110001 China; 3 Medical Administration Department, the First Hospital of China Medical University, Shenyang, 110001 China; 4 Department of Vascular and Thyroid Surgery, the First Hospital of China Medical University, Shenyang, 110001 China

**Keywords:** Toll-like receptor 4, Matrix metalloproteinase 9, Aortic aneurysm, Polymorphism, Interaction

## Abstract

**Background:**

A cross-talk between Toll-like receptor 4 (TLR4) and matrix metalloproteinase 9 (MMP9) plays a vital role in aortic pathophysiology. The objective of this study was to evaluate the interactions between TLR4 and MMP9 polymorphisms in the risk of aortic aneurysm (AA) and its subtypes.

**Methods:**

KASP method was used to detect polymorphisms of TLR4 (rs11536889 and rs1927914) and MMP9 (rs17576) in 472 AA patients and 498 controls. According to location and size, AA patients were further classified into abdominal AA (AAA), thoracic AA (TAA), and large AA (>5.0 cm), small AA(≤5.0 cm), respectively.

**Results:**

The significant interaction effect of TLR4rs1927914 with MMP9rs17576 polymorphisms was observed for the risk of TAA (*P*_*interaction*_ = 0.038, OR = 6.186) and large AA (*P*_*interaction*_ = 0.044, OR = 5.892). There were epistatic effects between TLR4rs1927914 and MMP9rs17576 polymorphisms on the risk of overall AA, AAA, TAA and large AA when they were present together. Moreover, the cumulative effects of the pairwise interaction TLR4rs1927914-MMP9rs17576 were associated with an increased risk of overall AA (*P*_*trend*_ = 0.032) and AAA (*P*_*trend*_ = 0.031).

**Conclusions:**

The novel interaction between TLR4rs1927914 and MMP9rs17576 polymorphisms could increase the risk of AA disease or its subtypes by exerting epistatic and cumulative effects.

**Electronic supplementary material:**

The online version of this article (10.1186/s12872-019-1049-8) contains supplementary material, which is available to authorized users.

## Background

Aortic aneurysm (AA) is a life-threatening complex disease with uncertain etiology and can occur in the abdominal or thoracic section of aorta. Although abdominal aortic aneurysm (AAA) and thoracic aortic aneurysm (TAA) have heterogeneity in their inheritance, incidence and distribution along aorta, they share some similar pathological states and histological phenotypes, including inflammatory cell infiltration and extracellular matrix (ECM) degradation [1, 2]. AA size has been reported to be an important independent predictor of perioperative morbidity and mortality, and special attention is deserved once maximal aortic diameter approaches the 5.0–5.5 cm threshold, which is perceived as an abrupt transition and increases the rupture risk of an aneurysm [3, 4]. There are several confounding factors associated with AA, including age, gender, hypertension and dyslipidemia [5, 6]. As for diabetes, whether it has a protective or risk effect on AA is far from definitive [7, 8]. Accumulating evidence supports an important role of genetic susceptibility in the initiation and development of AA. The activity and function of genes can be modulated by single nucleotide polymorphisms (SNPs), which may have impacts in amino acid substitutions changing protein function, in coding regions disturbing mRNA stability sequences or in promoter regions altering transcription factor binding motifs [9]. Although a large number of literatures have studied the association of genetic polymorphisms with AA, the responsible genes remain largely unidentified.

Inflammation and degradation of aortic media are considered mainly responsible for aortic expansion and aneurysm formation [10]. Toll-like receptor 4 (TLR4) is one of the most well-characterized inflammation-related molecules, and its signaling plays a key role in triggering inflammatory response in the aortic wall [9, 10]. A growing number of cell and animal experiments have revealed the importance of TLR4 pathway in AA formation [10–12]. TLR4 dysfunction caused by SNPs may change the ligand binding and balance between pro- and anti-inflammatory cytokines, and studies have demonstrated a functional significance of TLR4 polymorphisms in modulating the risk of some inflammatory diseases [13–15]. However, there were lacking researches on the relationship between TLR4 polymorphisms and AA risk. Recent studies reported that TLR4 took part in vascular homeostasis through a cross-talk with other signaling pathways, including ECM degradation pathway [11, 16]. Because of its capacity of degrading multiple extracellular components in aortic wall, matrix metalloproteinase 9 (MMP9) has been involved in aneurysm formation [2, 17]. A number of MMP9 polymorphisms have been investigated to discuss their associations with the risk of TAA or AAA, but often limited to a single locus, which seems insufficient as the genetic basis in predicting the risk of AA. However, multigene SNP-SNP interactions may amplify the main effect of individual SNP in complex diseases and enhance the predictive power [18].

Consequently, in this study, we firstly focused on the two- and three-dimensional interactions among TLR4 and MMP9 polymorphisms for their potential roles in susceptibility to AA and its subtypes stratified by location and size. We hoped to find the combined effects of gene-gene polymorphisms that might predict the risk of this disease and provide the evidence for the early detection of AA.

## Methods

### Study population

The study was approved by the Ethics Committee of the First Hospital of China Medical University (Shenyang, China). Written informed consent was obtained from each participant. A total of 472 AA patients (including 212 AAA patients, 216 TAA patients, 44 TAA combined AAA patients) and 498 healthy controls were recruited from the First Hospital of China Medical University between September 2016 and November 2017. The patients were enrolled from the cardiac or vascular surgery department of our hospital in which hospitalized patients received the treatment of AA. The control subjects were recruited from the physical examination center of our hospital and frequency-matched to AA cases on the basis of gender and age (±5 years). That means an individual in the control group can be matched to AA case so long as it has the same gender and neither the age differences between the control and case are more than 5 years. The diagnosis of all patients was based on the computed tomography angiography (CTA). Exclusion criteria included the subjects with coronary heart disease, congenital heart disease, severe vascular stenosis, autoimmune disease, severe organ failure, infectious disease, hematological system disease and malignant tumor. Available maximal aortic diameter of AA patients was obtained from CTA and all participants had their baseline characteristics recorded, as shown in Table 1. According to the maximal aortic diameter, AA patients were further classified into large AA (>5.0 cm) and small AA (≤5.0 cm). A 5-ml fasting venous blood sample was taken from each subject for DNA isolation.

**Table 1:** Baseline characteristics of the study participants

Variable	CON	AA
	*n* = 498	*n* = 472
Age, years	60.55 ± 12.61	60.97 ± 12.62
Male, n (%)	355(71.3%)	342(72.5%)
Hypertension, n (%)	214(43.0%)	327(69.3%)^*^
SBP, mmHg	136.42 ± 20.61	153.33 ± 26.61^*^
DBP, mmHg	78.70 ± 12.06	86.89 ± 16.48^*^
Diabetes, n (%)	55(11.0%)	125(26.5%)^*^
FPG, mmol/L	5.67 ± 1.49	6.36 ± 1.88^*^
Dyslipidemia, n (%)	203(40.8%)	262(55.5%)^*^
TC, mmol/L	4.56 ± 1.10	4.96 ± 0.91^*^
TG, mmol/L	4.56 ± 1.10	4.96 ± 0.91^*^
HDL-C, mmol/L	1.29 ± 0.33	1.10 ± 0.37^*^
LDL-C, mmol/L	2.87 ± 0.95	3.14 ± 0.78^*^
AA type		
AAA(%)	–	212(44.9%)
TAA(%)	–	216(45.8%)
TAAA(%)	–	44(9.3%)
Max. aortic diameter, cm	–	5.03 ± 1.48
>5.0 cm	–	160(33.9%)
≤ 5.0 cm	–	272(57.6%)
Missing	–	40(8.5%)

^*^P < 0.05, AA vs. CON; *AA* aortic aneurysm, *CON* control

Hypertension was defined as having a systolic blood pressure (SBP) ≥ 140 mmHg and/or having a diastolic blood pressure (DBP) ≥ 90 mmHg and/or being under antihypertensive treatment. Diabetes was defined as fasting serum glucose (FPG) ≥ 7 mmol/L and/or being on treatment for diabetes. Dyslipidemia was defined as serum total cholesterol (TC) ≥ 6.22 mmol/L, or triglyceride (TG) ≥ 2.26 mmol/L, or high-density lipoprotein cholesterol (HDL-C) < 1.03 mmol/L, or low-density lipoprotein cholesterol (LDL-C) ≥ 4.14 mmol/L and/or under taking hypolipidemic drugs.

### SNP selection and genotyping assay

According to previous description [19], we adopted a two-step method to select tag-SNPs for the genes of interest. In brief, tag-SNPs were chosen in the combinations provided by data from the HapMap database (http://www.HapMap.org) and Haploview software 4.2 (http://www.broadinstitute.org/mpg/haploview). Then, SNPinfo Web Server (https://snpinfo.niehs.nih.gov/) was used to predict their potential functions. Finally, two TLR4 SNPs (rs11536889 in the 3′-untranslated region (UTR), rs1927914 in the promoter region) and one MMP9 SNP (rs17576 in the exon 6) were chosen in this study.

Genomic DNA of each subject was extracted from a blood clot by a routine phenol-chloroform approach and then diluted to a working concentration of 50 ng/μL for genotyping. All samples were randomly placed in the 384-well plates and blinded for disease status. SNP genotyping was performed by Baygene Biotechnology Company Limited (Shanghai, China) using the KASP method with SNPLine platform (LGC, United Kingdom). Additionally, we randomly selected samples for repeated assays and the results were 100% consistent.

### Statistical analysis

All the statistical analyses were performed with SPSS 17.0 software (SPSS Inc., Chicago, IL, United States). Differences of baseline characteristics between case and control groups were estimated by independent-sample t-test or χ^2^ test as appropriate. The two- or three-dimensional SNP-SNP interaction effects were assessed using multivariate logistic regression analysis by comparing the model that only involved the main effects with the full model that also contained the interaction term. Associations were calculated by odds ratios (ORs) and 95% confidence intervals (95%CIs) with adjustments for age, gender, hypertension, diabetes and dyslipidemia. As for the cumulative effect, linear regression model was used to explore the trends with an increasing number of risk genotypes. A two-sided *P*<0.05 was considered statistically significant. The Bonferroni correction was used to adjust *P* values for multiple tests as needed. Additionally, this study defined the dominant and recessive genetic models as heterozygote+homozygote variant vs. homozygote wild and homozygote variant vs. heterozygote+homozygote wild, respectively.

## Results

### Main effect of individual TLR4 and MMP9 polymorphisms

The distributions of studied genotypes of TLR4rs11536889, rs1927914 and MMP9rs17576 in the controls followed Hardy-Weinberg equilibrium (HWE) (*P* > 0.05) (Additional file 1: Table S1). As shown in Additional file 2: Table S2, there were no significant associations of TLR4 rs11536889 and rs1927914 polymorphisms with the risk of AA and its subtypes (*P* > 0.05) in the general and subgroup analysis stratified by AA location and size. MMP9rs17576 AA genotype was related to a higher risk of overall AA (OR = 1.897, *P* = 0.015), AAA (OR = 2.291, *P* = 0.007) or small AA (OR = 1.977, *P* = 0.024) compared with GG genotype. However, no significant link between MMP9rs17576 polymorphisms and the risk of TAA and large AA was observed (*P* > 0.05).

### Two-way interactions between polymorphisms of TLR4 and MMP9

In the two-way interaction analyses with a combined genotype containing the most common SNP for each gene, the most significant interaction was between dominant genetic model of TLR4rs1927914 and recessive genetic model of MMP9rs17576 (Table 2). This interaction was linked to an increased risk of TAA (*P*_*interaction*_ = 0.038, OR = 6.186) and large AA (*P*_*interaction*_ = 0.044, OR = 5.892). In contrast, no statistically significant interaction was found among other SNP-SNP interactions (*P*_*interaction*_ > 0.05).

**Table 2:** Two-way interactions between TLR4 and MMP9 polymorphisms in the risk of AA^a^

TLR4	Genotypes	Number of Participants	MMP9rs17576			
			GG + GA	AA	GG	AA+GA
AA vs. CON						
rs11536889	GG	No. of controls/cases	290/273	18/29	161/148	147/154
		OR(95% CI)	1.0(ref.)	0.917(0.690–1.220)	1.0(ref.)	1.140(0.830–1.566)
	GC + CC	No. of controls/cases	154/133	7/16	86/75	75/74
		OR(95% CI)	0.917(0.690–1.220)	2.428(0.984–5.993)	0.949(0.648–1.390)	1.073(0.726–1.587)
			*P*_*interaction*_ = 0.430, OR = 1.559(0.517–4.701)		*P*_*interaction*_ = 0.979, OR = 1.007(0.582–1.744)	
	GG + GC	No. of controls/cases	419/381	25/42	232/211	212/212
		OR(95% CI)	1.0(ref.)	1.848(1.105–3.090)	1.0(ref.)	1.100(0.842–1.435)
	CC	No. of controls/cases	25/25	0/3	15/12	10/16
		OR(95% CI)	1.100(0.621–1.947)	NA	0.880(0.403–1.922)	1.759(0.781–3.962)
			*P*_*interaction*_ = NA, OR = NA		*P*_*interaction*_ = 0.289, OR = 1.842(0.595–5.700)	
rs1927914	TT	No. of controls/cases	168/145	12/13	104/81	76/77
		OR(95% CI)	1.0(ref.)	1.255(0.555–2.837)	1.0(ref.)	1.301(0.846–1.999)
	TC + CC	No. of controls/cases	268/255	14/32	138/144	144/143
		OR(95% CI)	1.103(0.833–1.460)	2.648(1.360–5.156)	1.340(0.923–1.945)	1.275(0.880–1.848)
			*P*_*interaction*_ = 0.274, OR = 1.797(0.629–5.131)		*P*_*interaction*_ = 0.253, OR = 0.729(0.424–1.253)	
	TT + TC	No. of controls/cases	365/332	23/37	205/187	183/182
		OR(95% CI)	1.0(ref.)	1.769(1.029–3.039)	1.0(ref.)	1.090(0.820–1.450)
	CC	No. of controls/cases	71/68	3/8	37/38	37/38
		OR(95% CI)	1.053(0.731–1.516)	2.932(0.771–11.143)	1.126(0.687–1.845)	1.126(0.687–1.845)
			*P*_*interaction*_ = 0.561, OR = 1.549(0.355–6.766)		*P*_*interaction*_ = 0.804, OR = 0.915(0.453–1.846)	
AA type						
AAA vs. CON						
rs11536889	GG	No. of controls/cases	290/123	18/16	161/68	147/71
		OR(95% CI)	1.0(ref.)	2.096(1.035–4.244)	1.0(ref.)	1.144(0.766–1.707)
	GC + CC	No. of controls/cases	154/56	7/9	86/34	75/31
		OR(95% CI)	0.857(0.591–1.243)	3.031(1.104–8.323)	0.936(0.575–1.525)	0.979(0.590–1.622)
			*P*_*interaction*_ = 0.298, OR = 1.972(0.549–7.086)		*P*_*interaction*_ = 0.927, OR = 1.034(0.506–2.111)	
	GG + GC	No. of controls/cases	419/171	25/23	232/97	212/97
		OR(95% CI)	1.0(ref.)	2.254(1.245–4.081)	1.0(ref.)	1.094(0.781–1.534)
	CC	No. of controls/cases	25/8	0/2	15/5	10/5
		OR(95% CI)	0.784(0.347–1.773)	NA	0.797(0.282–2.254)	1.196(0.398–3.590)
			*P*_*interaction*_ = NA, OR = NA		*P*_*interaction*_ = 0.677, OR = 1.386(0.298–6.443)	
rs1927914	TT	No. of controls/cases	168/62	12/10	104/38	76/34
		OR(95% CI)	1.0(ref.)	2.903(1.325–6.361)	1.0(ref.)	1.224(0.707–2.120)
	TC + CC	No. of controls/cases	268/114	14/15	138/65	144/64
		OR(95% CI)	1.153(0.801–1.660)	2.589(0.929–5.489)	1.289(0.802–2.071)	1.216(0.757–1.954)
			*P*_*interaction*_ = 0.829, OR = 0.876(0.265–2.898)		*P*_*interaction*_ = 0.468, OR = 0.772(0.384–1.553)	
	TT + TC	No. of controls/cases	365/144	23/23	205/84	183/83
		OR(95% CI)	1.0(ref.)	2.535(1.378–4.662)	1.0(ref.)	1.107(0.770–1.591)
	CC	No. of controls/cases	71/32	3/2	37/19	37/15
		OR(95% CI)	1.142(0.721–1.809)	1.690(0.279–10.218)	1.253(0.682–2.303)	0.989(0.516–1.898)
			*P*_*interaction*_ = 0.476, OR = 0.490(0.069–3.476)		*P*_*interaction*_ = 0.429, OR = 0.694(0.280–1.717)	
TAA vs. CON						
rs11536889	GG	No. of controls/cases	290/125	18/10	161/63	147/72
		OR(95% CI)	1.0(ref.)	1.289(0.579–2.871)	1.0(ref.)	1.252(0.835–1.877)
	GC + CC	No. of controls/cases	154/66	7/5	86/35	75/36
		OR(95% CI)	0.994(0.696–1.420)	1.657(0.516–5.321)	1.040(0.638–1.696)	1.227(0.749–2.008)
			*P*_*interaction*_ = 0.667, OR = 1.370(0.326–5.764)		*P*_*interaction*_ = 0.828, OR = 0.926(0.463–1.851)	
	GG + GC	No. of controls/cases	419/174	25/14	232/91	212/97
		OR(95% CI)	1.0(ref.)	1.349(0.685–2.656)	1.0(ref.)	1.166(0.829–1.641)
	CC	No. of controls/cases	25/17	0/1	15/7	10/11
		OR(95% CI)	1.637(0.863–3.109)	NA	1.190(0.470–3.013)	2.804(1.152–6.829)
			*P*_*interaction*_ = NA, OR = NA		*P*_*interaction*_ = 0.280, OR = 2.037(0.561–7.405)	
rs1927914	TT	No. of controls/cases	168/72	12/2	104/38	76/36
		OR(95% CI)	1.0(ref.)	0.389(0.085–1.782)	1.0(ref.)	1.296(0.753–2.232)
	TC + CC	No. of controls/cases	268/117	14/13	138/61	144/69
		OR(95% CI)	1.019(0.717–1.447)	2.167(0.970–4.840)	1.210(0.750–1.952)	1.311(0.820–2.097)
			*P*_*interaction*_ = 0.038 (0.076^b^), OR = 6.186(1.103–34.690)		*P*_*interaction*_ = 0.609, OR = 0.836(0.421–1.660)	
	TT + TC	No. of controls/cases	365/159	23/11	205/83	183/87
		OR(95% CI)	1.0(ref.)	1.098(0.523–2.306)	1.0(ref.)	1.174(0.819–1.684)
	CC	No. of controls/cases	71/30	3/4	37/16	37/18
		OR(95% CI)	0.970(0.609–1.545)	3.061(0.677–13.834)	1.068(0.563–2.024)	1.202(0.647–2.230)
			*P*_*interaction*_ = 0.180, OR = 3.282(0.579–18.608)		*P*_*interaction*_ = 0.951, OR = 0.972(0.399–2.371)	
AA size						
large AA vs. CON						
rs11536889	GG	No. of controls/cases	290/91	18/12	161/57	147/46
		OR(95% CI)	1.0(ref.)	2.125(0.986–4.577)	1.0(ref.)	0.884(0.565–1.384)
	GC + CC	No. of controls/cases	154/45	7/6	86/29	75/22
		OR(95% CI)	0.931(0.620–1.399)	2.732(0.895–8.335)	0.952(0.567–1.599)	0.829(0.472–1.455)
			*P*_*interaction*_ = 0.613, OR = 1.437(0.353–5.842)		*P*_*interaction*_ = 0.901, OR = 1.051(0.478–2.309)	
	GG + GC	No. of controls/cases	419/124	25/18	232/79	212/63
		OR(95% CI)	1.0(ref.)	2.433(1.285–4.605)	1.0(ref.)	0.873(0.597–1.276)
	CC	No. of controls/cases	25/12	0/0	15/7	10/5
		OR(95% CI)	1.622(0.792–3.322)	NA	1.370(0.539–3.483)	1.468(0.487–4.426)
			*P*_*interaction*_ = 0.211, OR = 1.591(0.769–3.289)		*P*_*interaction*_ = 0.756, OR = 1.263(0.290–5.503)	
rs1927914	TT	No. of controls/cases	168/53	12/2	104/34	76/21
		OR(95% CI)	1.0(ref.)	0.528(0.115–2.436)	1.0(ref.)	0.845(0.455–1.570)
	TC + CC	No. of controls/cases	268/82	14/16	138/54	144/44
		OR(95% CI)	0.970(0.653–1.441)	3.623(1.659–7.910)	1.197(0.727–1.971)	0.935(0.559–1.562)
			*P*_*interaction*_ = 0.044(0.088^b^), OR = 5.892(1.048–33.115)		*P*_*interaction*_ = 0.881, OR = 0.942(0.431–2.059)	
	TT + TC	No. of controls/cases	365/113	23/15	205/72	183/56
		OR(95% CI)	1.0(ref.)	2.107(1.063–4.174)	1.0(ref.)	0.871(0.583–1.303)
	CC	No. of controls/cases	71/22	3/3	37/16	37/9
		OR(95% CI)	1.001(0.593–1.688)	3.230(0.643–16.227)	1.231(0.646–2.347)	0.693(0.319–1.505)
			*P*_*interaction*_ = 0.806, OR = 1.257(0.203–7.796)		*P*_*interaction*_ = 0.418, OR = 0.653(0.233–1.831)	
small AA vs. CON						
rs11536889	GG	No. of controls/cases	290/159	18/16	161/82	147/93
		OR(95% CI)	1.0(ref.)	1.621(0.805–3.267)	1.0(ref.)	1.242(0.857–1.801)
	GC + CC	No. of controls/cases	154/78	7/9	86/40	75/47
		OR(95% CI)	0.924(0.661–1.290)	2.345(0.857–6.416)	0.913(0.577–1.446)	1.230(0.784–1.932)
			*P*_*interaction*_ = 0.475, OR = 1.572(0.454–5.446)		*P*_*interaction*_ = 0.838, OR = 1.070(0.562–2.034)	
	GG + GC	No. of controls/cases	419/225	25/22	232/118	212/129
		OR(95% CI)	1.0(ref.)	1.639(0.904–2.972)	1.0(ref.)	1.196(0.876–1.634)
	CC	No. of controls/cases	25/12	0/3	15/4	10/11
		OR(95% CI)	0.894(0.441–1.813)	NA	0.524(0.170–1.615)	2.163(0.893–5.238)
			*P*_*interaction*_ = NA, OR = NA		*P*_*interaction*_ = 0.093, OR = 3.411(0.814–14.288)	
rs1927914	TT	No. of controls/cases	168/81	12/11	104/41	76/51
		OR(95% CI)	1.0(ref.)	1.901(0.805–4.493)	1.0(ref.)	1.702(1.026–2.825)
	TC + CC	No. of controls/cases	268/151	14/14	138/80	144/85
		OR(95% CI)	1.169(0.839–1.628)	2.074(0.944–4.555)	1.470(0.933–2.316)	1.497(0.955–2.348)
			*P*_*interaction*_ = 0.927, OR = 0.947(0.298–3.008)		*P*_*interaction*_ = 0.116, OR = 0.600(0.318–1.134)	
	TT + TC	No. of controls/cases	365/194	23/21	205/100	183/115
		OR(95% CI)	1.0(ref.)	1.718(0.927–3.183)	1.0(ref.)	1.288(0.922–1.799)
	CC	No. of controls/cases	71/38	3/4	37/21	37/21
		OR(95% CI)	1.007(0.655–1.549)	2.509(0.556–11.322)	1.164(0.647–2.091)	1.164(0.647–2.091)
			*P*_*interaction*_ = 0.629, OR = 1.508(0.284–8.002)		*P*_*interaction*_ = 0.561, OR = 0.782(0.342–1.791)	

^a^, *P* for association was adjusted by age, gender, hypertension, diabetes and dyslipidemia; ^b^, *P* values after Bonferroni correction; *AA* aortic aneurysm, *AAA* abdominal aortic aneurysm, *TAA* thoracic aortic aneurysm, *CON* control, *NA* not available

### Epistatic effects of two-way interactions between TLR4rs1927914 and MMP9rs17576

We further investigated the epistatic effects between pairs of interacting factors, as shown in Table 3 and Table 4. For TLR4rs1927914 and MMP9rs17576, TC + CC genotype at rs1927914 and AA genotype at rs17576 each conferred an increased TAA risk (OR = 6.329 and 2.322, respectively) and large AA (OR = 6.554 and 3.243, respectively), but only if they were both present; rs17576 AA genotype was correlated with an increased risk of overall AA (OR = 2.333) and AAA (OR = 2.519), but only in the presence of TC + CC genotype at rs1927914.

**Table 3:** Epistatic effect of pair-wise interacting factors of TLR4rs1927914 and MMP9rs17576 on overall AA risk^a^

Comparison	Subset	*P*	OR(95%CI)
TLR4rs1927914	MMP9rs17576		
TC + CC vs. TT	GG + GA	0.395	1.131(0.852–1.500)
	MMP9rs17576		
	AA	0.160	2.150(0.740–6.246)
MMP9rs17576	TLR4rs1927914		
AA vs. GG + GA	TT	0.531	1.301(0.571–2.964)
	TLR4rs1927914		
	TC + CC	0.011(0.022^b^)	2.333(1.213–4.488)

^a^, *P* for association was adjusted by age, gender, hypertension, diabetes and dyslipidemia; ^b^, *P* values after Bonferroni correction

**Table 4:** Epistatic effect of pair-wise interacting factors of TLR4rs1927914 and MMP9rs17576 on the risk of AA subtypes^a^

		AAA vs. CON		TAA vs. CON		large AA vs. CON		small AA vs. CON	
Comparison	Subset	*P*	OR(95%CI)	*P*	OR(95%CI)	*P*	OR(95%CI)	*P*	OR(95%CI)
TLR4rs1927914	MMP9rs17576								
TC + CC vs. TT	GG + GA	0.244	1.247(0.860–1.809)	0.940	0.986(0.692–1.406)	0.902	1.025(0.687–1.531)	0.357	1.170(0.838–1.633)
	MMP9rs17576								
	AA	0.686	1.266(0.403–3.985)	0.041(0.082^b^)	6.329(1.077–37.184)	0.032(0.064^b^)	6.554(1.172–36.649)	0.736	1.219(0.386–3.850)
MMP9rs17576	TLR4rs1927914								
AA vs. GG + GA	TT	0.072	2.258(0.929–5.489)	0.202	0.369(0.080–1.706)	0.436	0.542(0.116–2.528)	0.150	1.890(0.794–4.496)
	TLR4rs1927914								
	TC + CC	0.017(0.034^b^)	2.519(1.177–5.389)	0.038(0.076^b^)	2.322(1.047–5.150)	0.003(0.006^b^)	3.243(1.488–7.068)	0.136	1.795(0.832–3.872)

^a^, *P* for association was adjusted by age, gender, hypertension, diabetes and dyslipidemia; ^b^, *P* values after Bonferroni correction; *AA* aortic aneurysm, *AAA* abdominal aortic aneurysm, *TAA* thoracic aortic aneurysm, *CON* control

### Cumulative effect of the interacting factors of TLR4rs1927914 and MMP9rs17576

We also evaluated the cumulative effect among the polymorphism interaction between TLR4rs1927914 and MMP9rs17576. A dosage effect was significantly observed with an increase in the number of risk genotypes linked to an increased risk of overall AA (*P*_*trend*_ = 0.032) (Table 5) and AAA (*P*_*trend*_ = 0.031) (Table 6). However, other subtype of AA risk was not statistically increased while one or two mutation genotypes were present.

**Table 5:** Cumulative effect of the interacting factors of TLR4rs1927914-MMP9rs17576 on overall AA risk^a^

No. of interacting genotypes	Controls/cases	*P*	OR(95%CI)
TLR4rs1927914-MMP9rs17576			
0	168/145		1.0(ref.)
1	280/268	0.381	1.134(0.856–1.500)
2	14/32	0.005	2.609(1.336–5.097)
		*P*_*trend*_ = 0.032	

^a^, *P* for association was adjusted by age, gender, hypertension, diabetes and dyslipidemia

**Table 6:** Cumulative effect of the interacting factors of TLR4rs1927914-MMP9rs17576 on the risk of AA subtypes^a^

No. of interacting genotypes	AAA			TAA			large AA			small AA		
	Controls/cases	*P*	OR(95%CI)	Controls/cases	*P*	OR(95%CI)	Controls/cases	*P*	OR(95%CI)	Controls/cases	*P*	OR(95%CI)
TLR4rs1927914-MMP9rs17576												
0	168/62		1.0(ref.)	168/72		1.0(ref.)	168/53		1.0(ref.)	168/81		1.0(ref.)
1	280/124	0.170	1.293(0.896–1.865)	280/119	0.808	0.957(0.673–1.362)	280/84	0.001	1.002(0.673–1.492)	280/162	0.287	1.196(0.860–1.664)
2	14/15	0.014	2.736(1.222–6.128)	14/13	0.058	2.180(0.973–4.886)	14/16	0.004	3.234(1.456–7.184)	14/14	0.072	2.060(0.936–4.533)
		*P*_*trend*_ = 0.031			*P*_*trend*_ = 0.338			*P*_*trend*_ = 0.079			*P*_*trend*_ = 0.084	

^a^, *P* for association was adjusted by age, gender, hypertension, diabetes and dyslipidemia; *AA* aortic aneurysm, *AAA* abdominal aortic aneurysm, *TAA* thoracic aortic aneurysm

### Three-dimensional interactions among polymorphisms of TLR4 and MMP9 genes

We further examined potential three-dimensional interactions among TLR4rs11536889, rs1927914 and MMP9rs17576, however, no statistical significance among three-dimensional interactions was observed in relation to the risk of overall AA (Table 7) and its subtypes (Additional file 3: Table S3).

**Table 7:** The three-dimensional interactions of the MMP9rs17576-TLR4rs11536889-TLR4rs1927914 with overall AA risk^a^

SNP genotypes			*P*	OR(95%CI)
MMP9rs17576-TLR4rs11536889-TLR4rs1927914				
GG + GA	GG	TT		1(ref)
GG + GA	GG	TC + CC	0.294	1.230(0.836–1.810)
GG + GA	GC + CC	TT	0.594	1.130(0.721–1.773)
GG + GA	GC + CC	TC + CC	0.942	0.982(0.594–1.622)
AA	GG	TT	0.844	0.887(0.268–2.932)
AA	GG	TC + CC	0.013	2.710(1.232–5.959)
AA	GC + CC	TT	0.152	2.484(0.715–8.635)
AA	GC + CC	TC + CC	0.086	3.312(0.843–13.012)
			*P*_*interaction*_ = 0.696, OR = 0.629(0.061–6.451)	

^a^, *P* for association was adjusted by age, gender, hypertension, diabetes and dyslipidemia

## Discussion

Human genetic polymorphisms can be used to predict the presence of particular diseases. However, a substantial body of studies have concentrated on the identification of SNP responsible for disease risk, but they often found no or weak effects [20, 21]. Therefore, individual SNP association cannot adequately account for the causality of complex disease. Recently, researchers are focusing more attention on the potential combined interaction effects of SNPs in two or more loci, and the results have usually indicated a moderate or strong effect on disease risk [22, 23]. As the gate of inflammatory reaction, TLR4 can induce pathological aortic phenotype changes, such as outward vascular remodeling [24]. MMP9, as a multifunctional proteinase, is responsible for ECM degradation and weakening aortic wall [25]. Recently, Li et al. demonstrated the role of TLR4 signaling in the regulation of MMP9 expression in human aortic smooth muscle cells [26]. Qin et al. reported that the mice deficiency of TLR4 gene exhibited markedly decreased AngII-mediated AAA formation and MMP9 secretion, and TLR4 levels were elevated in human aneurysmal tissue [27]. These data suggested that TLR4 pathway linked to MMP9 appeared to have a key role in AA pathophysiology, but the gene association study was lacking. To the best of our knowledge, this study is the first to evaluate the interaction effects of two tag-SNP rs11536889 and rs1927914 in TLR4 and one tag-SNP rs17576 in MMP9 on the risk of AA and its subtypes in a Chinese Han population.

TLR4rs11536889 is located in the 3′-UTR of, where a genetic change can influence the translation of mRNA [28]. SNP rs1927914 locates in the 5′-UTR of TLR4 and may influence transcriptional factor binding site and regulate the promoter activity [14]. TLR4rs11536889 and rs1927914 polymorphisms have been reported to be correlated with various inflammatory diseases [29–31]. Furthermore, SNP rs17576 is a coding variant in exon 6 of MMP9 leading to an amino acid substitution, which might increase the enzyme activity of MMP9 [32]. Some researchers have reported that MMP9rs17576 polymorphisms were associated with lung cancer [33], left ventricular dysfunction in coronary artery disease [34] and carotid artery intima-media thickness [35]. However, none of above three SNPs has been studied in AA disease by now. Compared with a SNP, a combination of two or more SNPs could generate synergistic or antagonistic effects, which would change the susceptibility to disease [36, 37]. Individually, TLR4rs11536889, rs1927914 and MMP9rs17576 had no effects on the risk of TAA and large AA. In the analysis of two-way interaction, the paired TLR4rs1927914-MMP9rs17576 polymorphism demonstrated obvious interaction effects with ORs of 6.186 and 5.892 for the risk of TAA and large AA, respectively. Our findings indicated their strong synergistic effects, which could alter the risk of an individual towards AA. This may partly interpret the epigenetic heritability loss of AA risk and indicate a novel insight into the multifactorial etiology of AA risk concerning inflammation-related gene TLR4 and ECM degradation gene MMP9 pathways. Maximal aortic diameter has been found to link directly to the risk of aortic complications [38]. The phenomenon of an effect modification by maximal aortic diameter perceived in the TLR4 and MMP9 interaction may provide a crucial hint to help avoid AA complications by preventing diameter growth in susceptible people.

The epistatic effect of two or more genes can explain the missing heritability of many diseases, which is usually underestimated or even ignored [39]. The epistasis was more prominent than a single susceptible gene in terms of main effect, which implied the multipart interaction effect [23]. Many studies have suggested that epistatic gene-gene interactions offered susceptibility to a variety of cancer [40, 41]. In fact, as a complex disease, only in rare cases does AA seem to be monogenic and, generally, multiple genes are involved in AA occurrence. However, different types of AA may have different genetic background and show varying sensitivity to the effects of genetic polymorphisms. In the present study, the most significant epistatic effect was between TLR4rs1927914 and MMP9rs17576. Both rs1927914 TC + CC genotype and rs17576 AA genotype were related to an increased risk of TAA and large AA. Moreover, AA genotype of rs17576 conferred an increased risk of overall AA and AAA in the presence of TC + CC genotype of rs1927914 even after Bonferroni correction, which was a strict method for multiple comparisons. These observations revealed that the obviously epistatic effect of TLR4rs1927914 and MMP9rs17576 on the pathogenesis of AA might rely on the presence of the other SNP in each pair-wise interaction. However, the direct evidence for a specific functional association between TLR4 and MMP9 polymorphisms is scarce. Only one study by Ruvolo et al. in 2014 reported that TLR4rs4986790 polymorphism confers a higher susceptibility for TAA and it represents, together with ACErs1799752, MMP9rs3918242 and MMP2rs2285053 SNPs, an independent TAA risk factor [42]. Genetic variants of any gene within these networks could have a potential influence on the action of other genes and thus disrupt the balance of homeostasis.

In this study, the cumulative effect was found to be altered in overall AA and AAA. The dose effects suggested that overall AA risk was significantly increased when one or two risk genotypes were present (OR = 1.134 and 2.609, respectively). Meanwhile, AAA risk was significantly increased, while one or two risk genotypes were present (OR = 1.293 and 2.736, respectively). The cumulative effect of the two SNPs was stronger than an individual SNP effect, which is indicative of a true interaction. However, we further analyzed the three-dimensional interaction effect of TLR4rs11536889-TLR4rs1927914-MMP9rs17576 on the risk of AA and its subtypes, but no interaction effect was shown among them. This may possibly attribute to our relatively small sample size and some scarce genotypes.

Our study has several limitations. First, this study was performed among a preselected population, thus the results may be affected by selection bias. Second, the sample size was relatively small and should be enlarged to confirm the results of the interaction effects, especially for rare genotypes. Third, complete lifestyle information (i.e. smoking and drinking status) was lost, precluding their use as potential environmental factors in our multivariate logistic regression. In addition, since we only tested two TLR4 SNPs and one MMP9 SNP in this study, comprehensive studies involving more functional SNPs should be conducted to perform a multiple testing correction in the future.

## Conclusions

In summary, we discovered novel SNP interactions among TLR4 and MMP9 which modified the AA risk, and our study was the first to show that the pairwise interacting TLR4rs1927914-MMP9rs17576 combinations were related to increased TAA and large AA risk. Furthermore, TLR4rs1927914 and MMP9rs17576 polymorphisms showed an epistatic effect when present together on the risk of AA and its subtypes, and these two-way interactions had dose effects on overall AA and AAA risk. TLR4rs1927914 might have greater efficiency when combined with MMP9rs17576 for AA risk than a single SNP on its own. Further functional and molecular experiments should deserve special attention to clarify the interaction effect of susceptible genes, which could provide an important clue for the etiology and perhaps effective treatment of AA.

## Additional files

Additional file 1: **Table S1.** The genotype frequencies and HWE in this study. (DOCX 16 kb)

Additional file 2: **Table S2.** The association of TLR4 and MMP9 polymorphisms with the risk of AA and its subtypes^a^. (DOCX 18 kb)

Additional file 3: **Table S3.** The three dimensions interactions of the MMP9rs17576-TLR4rs11536889-TLR4rs1927914 with the risk of AA subtypes^a^. (DOCX 19 kb)

## Abbreviations

AA: Aortic aneurysm
AAA: Abdominal aortic aneurysm
CI: Confidence interval
CTA: Computed tomography angiography
DBP: Diastolic blood pressure
ECM: Extracellular matrix
FPG: Fasting serum glucose
HDL-C: High-density lipoprotein cholesterol
HWE: Hardy-Weinberg equilibrium
LDL-C: Low-density lipoprotein cholesterol
MMP9: Matrix metalloproteinase 9
OR: Odds ratio
SBP: Systolic blood pressure
SNP: Single nucleotide polymorphism
TAA: Thoracic aortic aneurysm
TC: Total cholesterol
TG: Triglyceride
TLR4: Toll-like receptor 4
UTR: Untranslated region
