# Supplementary material for: The interaction effects between TLR4 and MMP9 gene polymorphisms contribute to aortic aneurysm risk in a Chinese Han population
Source: BMC Cardiovasc Disord. 2019 Mar 29;19:72. doi: 10.1186/s12872-019-1049-8 (PMC6439981; doi:10.1186/s12872-019-1049-8)
Supplement: Supplementary file 1 — Table S1. The genotype frequencies and HWE in this study. (DOCX 16 kb) [file 12872_2019_1049_MOESM1_ESM.docx]

| Table S1. The genotype frequencies and HWE in this study. | | | |
| --- | --- | --- | --- |
| Genotype | AA | CON | *P* Value of HWE |
| rs11536889(G＞C) |  |  | 0.113 |
| GG (%) | 310(66.5%) | 324(65.5%) |  |
| GC (%) | 127(27.3%) | 146(29.5%) |  |
| CC (%) | 29(6.2%) | 25(5.0%) |  |
| rs1927914(T＞C) |  |  | 0.137 |
| TT (%) | 165(35.9%) | 190(39.3%) |  |
| TC (%) | 219(47.6%) | 214(44.2%) |  |
| CC (%) | 76(16.5%) | 80(16.5%) |  |
| rs17576(G＞A) |  |  | 0.105 |
| GG (%) | 226(49.7%) | 249(52.8%) |  |
| GA (%) | 184(40.4%) | 197(41.7%) |  |
| AA (%) | 45(9.9%) | 26(5.5%) |  |
|  |  |  |  |

AA, aortic aneurysm; CON, control; HWE, Hardy-Weinberg equilibrium.
