# Supplementary material for: The interaction effects between TLR4 and MMP9 gene polymorphisms contribute to aortic aneurysm risk in a Chinese Han population
Source: BMC Cardiovasc Disord. 2019 Mar 29;19:72. doi: 10.1186/s12872-019-1049-8 (PMC6439981; doi:10.1186/s12872-019-1049-8)
Supplement: Supplementary file 2 — Table S2. The association of TLR4 and MMP9 polymorphisms with the risk of AA and its subtypesa. (DOCX 18 kb) [file 12872_2019_1049_MOESM2_ESM.docx]

| Table S2. The association of TLR4 and MMP9 polymorphisms with the risk of AA and its subtypes^a^. | | | | | | | | | | | |
| --- | --- | --- | --- | --- | --- | --- | --- | --- | --- | --- | --- |
|  | AA vs. CON | | | AAA vs. CON | | TAA vs. CON | | large AA vs. CON | | small AA vs. CON | |
|  | *P* | | OR(95%CI) | *P* | OR(95%CI) | *P* | OR(95%CI) | *P* | OR(95%CI) | *P* | OR(95%CI) |
| rs11536889 | | |  |  |  |  |  |  |  |  |  |
| GG |  | |  |  |  |  |  |  |  |  |  |
| GC | 0.660 | | 1.074(0.783-1.473) | 0.856 | 0.963(0.645-1.439) | 0.692 | 1.090(0.712-1.668) | 0.504 | 0.868(0.574-1.314) | 0.549 | 0.903(0.646-1.262) |
| CC | 0.366 | | 1.340(0.711-2.525) | 0.440 | 1.397(0.598-3.262) | 0.136 | 1.771(0.835-3.754) | 0.308 | 1.465(0.704-3.049) | 0.687 | 1.144(0.594-2.202) |
| rs1927914 | | |  |  |  |  |  |  |  |  |  |
| TT | |  |  |  |  |  |  |  |  |  |  |
| TC | | 0.593 | 1.089(0.797-1.488) | 0.671 | 1.090(0.733-1.622) | 0.813 | 1.051(0.695-1.589) | 0.337 | 1.217(0.815-1.818) | 0.323 | 1.181(0.849-1.644) |
| CC | | 0.915 | 1.024(0.662-1.585) | 0.772 | 1.083(0.630-1.864) | 0.768 | 1.091(0.611-1.950) | 0.728 | 1.102(0.638-1.904) | 0.832 | 1.050(0.669-1.649) |
| rs17576 | |  |  |  |  |  |  |  |  |  |  |
| GG | |  |  |  |  |  |  |  |  |  |  |
| GA | | 0.869 | 1.023(0.781-1.341) | 0.678 | 0.928(0.650-1.323) | 0.324 | 1.186(0.845-1.666) | 0.108 | 0.722(0.485-1.074) | 0.268 | 1.196(0.872-1.641) |
| AA | | 0.015(0.045^b^) | 1.897(1.132-3.177) | 0.007(0.021^b^) | 2.291(1.253-4.190) | 0.243 | 1.501(0.759-2.969) | 0.082 | 1.801(0.928-3.496) | 0.024(0.072^b^) | 1.977(1.093-3.575) |
|  | |  |  |  |  |  |  |  |  |  |  |

^a^, *P* for association was adjusted by age, gender, hypertension, diabetes and dyslipidemia; ^b^, *P* values after Bonferroni correction; AA, aortic aneurysm; AAA, abdominal aortic aneurysm; TAA, thoracic aortic aneurysm; CON, control.
