# Supplementary material for: The interaction effects between TLR4 and MMP9 gene polymorphisms contribute to aortic aneurysm risk in a Chinese Han population
Source: BMC Cardiovasc Disord. 2019 Mar 29;19:72. doi: 10.1186/s12872-019-1049-8 (PMC6439981; doi:10.1186/s12872-019-1049-8)
Supplement: Supplementary file 3 — Table S3. The three dimensions interactions of the MMP9rs17576-TLR4rs11536889-TLR4rs1927914 with the risk of AA subtypesa. (DOCX 19 kb) [file 12872_2019_1049_MOESM3_ESM.docx]

| Table S3. The three dimensions interactions of the MMP9rs17576-TLR4rs11536889-TLR4rs1927914 with the risk of AA subtypes^a^. | | | | | | | | | | | | |  |  | |  |  | | |
| --- | --- | --- | --- | --- | --- | --- | --- | --- | --- | --- | --- | --- | --- | --- | --- | --- | --- | --- | --- |
| SNP genotypes | |  | |  | AAA vs. CON | | | | TAA vs. CON | | | | large AA vs. CON | | | small AA vs. CON | | | |
|  | |  | |  | *P* | | OR(95%CI) | | *P* | OR(95%CI) | | | *P* | OR(95%CI) | | *P* | OR(95%CI) | | |
| MMP9rs17576-TLR4rs11536889-TLR4rs1927914 | | | | |  | |  | |  |  | | |  |  | |  |  | | |
| GG+GA | | GG | | TT |  | | 1(ref) | |  | 1(ref) | | |  | 1(ref) | |  | 1(ref) | | |
| GG+GA | | GG | | TC+CC | 0.874 | | 1.040（0.642-1.682） | | 0.128 | 1.495(0.890-2.511) | | | 0.871 | 0.957(0.563-1.628) | | 0.173 | 1.382(0.868-2.200) | | |
| GG+GA | | GC+CC | | TT | 0.482 | | 0.811（0.452-1.455） | | 0.088 | 1.658(0.928-2.962) | | | 0.831 | 0.934(0.501-1.743) | | 0.402 | 1.258(0.735-2.155) | | |
| GG+GA | | GC+CC | | TC+CC | 0.824 | | 0.931（0.498-1.742） | | 0.921 | 1.035(0.524-2.045) | | | 0.637 | 0.845(0.419-1.702) | | 0.865 | 1.054(0.576-1.929) | | |
| AA | | GG | | TT | 0.966 | | 1.031（0.251-4.241） | | 0.476 | 0.458(0.054-3.914) | | | NA | NA | | 0.411 | 1.667(0.493-5.634) | | |
| AA | | GG | | TC+CC | 0.023 | | 2.844（1.153-7.013） | | 0.057 | 2.625(0.972-7.086) | | | 0.011 | 3.360(1.320-8.553) | | 0.074 | 2.333(0.921-5.913) | | |
| AA | | GC+CC | | TT | 0.030 | | 4.211（1.152-15.386） | | 0.847 | 0.802(0.085-7.524) | | | 0.630 | 1.540(0.266-8.918) | | 0.065 | 3.500(0.926-13.224) | | |
| AA | | GC+CC | | TC+CC | 0.614 | | 1.604（0.256-10.061） | | 0.069 | 4.278(0.894-20.470) | | | 0.077 | 4.107(0.860-19.611) | | 0.315 | 2.333(0.447-12.167) | | |
|  | |  | |  | *P_interaction_*=0.178, OR=0.141(0.008-2.435) | | | | *P_interaction_*=0.688, OR=2.087(0.057-76.133) | | | | *P_interaction_* =NA, OR=NA | | | *P_interaction_* =0.846, OR=0.773(0.058-10.344) | | | |
|  | |  | | |  | |  |  | |  |  | | |  | |  |  |  | |

^a^, *P* for association was adjusted by age, gender, hypertension, diabetes and dyslipidemia; AA, aortic aneurysm; AAA, abdominal aortic aneurysm; TAA, thoracic aortic aneurysm; CON, control; NA, not available.
